# A systematic review of non-pharmacological interventions used for pain relief after orthopedic surgical procedures

**DOI:** 10.3892/etm.2020.9163

**Published:** 2020-09-01

**Authors:** Meifen Fan, Zheying Chen

**Affiliations:** Department of Operating Room, Renji Hospital, School of Medicine, Shanghai Jiao Tong University, Shanghai 200127, P.R. China

**Keywords:** pain, complementary therapy, surgery, music, relaxation therapy

## Abstract

The purpose of the present review was to evaluate the available evidence on the efficacy of various non-pharmacological interventions to relieve pain after orthopedic surgical procedures. An electronic search of the PubMed, Embase and Cochrane library databases was performed to retrieve studies of all types assessing the role of non-pharmacological interventions for pain relief after orthopedic surgical procedures. The included studies were required to assess pain outcomes using a validated measurement index, such as the Visual Analog Scale. The quality of randomized control trials (RCTs) was assessed using the Cochrane tool, while the ROBINS-I tool was used for non-RCTs. A total of five studies were included, namely three RCTs and two non-RCTs. The included studies used relaxation therapy, guided imagery, music and audio-visual distraction for pain management. There was considerable heterogeneity concerning study participants and types of intervention, which precluded a meta-analysis. Overall, all studies reported a significant beneficial effect of non-pharmacological interventions for pain relief. To conclude, current evidence from a limited number of studies indicates there may be a potential role of non-pharmacological interventions, including relaxation therapy, guided imagery, music and audio-visual distraction, in pain management of patients after orthopedic surgery. Owing to considerable heterogeneity and risk of bias in the included studies, strong conclusions cannot be drawn. Further high-quality RCTs assessing the role of such non-pharmacological techniques of pain management are required to strengthen the current evidence.

## Introduction

Orthopedic procedures are known to be painful to patients (1). In a comparison of pain intensity of surgical procedures, 22 of the 40 medical procedures with the highest pain level were orthopedic or trauma procedures of the extremities (2). Pain control is an important element of post-operative care as severe postoperative pain may increase the risk of complications, progress to persistent pain states and also delay rehabilitation (3). Post-operative pain may also increase overall healthcare costs due to a prolonged hospital stay and elevated rates of hospital readmission (4). Clinicians have reportedly used various means of pain control, including opioids, non-steroidal anti-inflammatory drugs (NSAIDs), local anesthetic infiltration and nerve blocks, for managing the analgesic requirements after orthopedic surgery (5). It is not surprising that orthopedic surgeons prescribe more opioids than any other surgical division (6).

A vital constituent of the pain management team is orthopedic nurses who spend considerable post-operative time with patients. Nursing personnel have an important role in patient preparation and management of the patients' pain and anxiety (7). They are not only responsible for the prescribed drug administration but may also support pain management by a variety of complementary therapies (8,9). Several studies and reviews have assessed the role of complementary therapy, including listening to music, media distraction, relaxation therapy and guided imagery in reducing procedural or post-operative pain in a variety of patients (7-13). However, the literature on the use of such complementary therapies for orthopedic patients is limited. There is a requirement to provide evidence on this subject, e.g. by a pooled data synthesis, to guide nursing personnel on the exact role of these therapies in orthopedic patients. Therefore, the purpose of the present review was to assess the evidence on the efficacy of various non-pharmacological interventions to relieve pain after orthopedic procedures.

## Materials and methods

### 

#### Search strategy

An electronic search of the PubMed, Embase and Cochrane library databases was performed by two independent reviewers (MF and ZC). The last search was carried out on 1^st^ July 2019. The following terms were employed for theliterature search: ‘orthopedics’, ‘wounds’, ‘injuries’, ‘fracture’, ‘music’, ‘video’, ‘guided imagery’, ‘relaxation’ and ‘breathing exercises’. Further details regarding the search are outlined in Table SI. References of included studies were screened for the identification of any further relevant trials. The search results of all databases were initially evaluated by their titles and abstracts. Relevant articles identified by the initial screening were further scanned by their full-texts for inclusion in the present review. Disagreements between the two reviewers were resolved by discussion. This review was conducted following the guidelines of the Preferred Reporting Items for Systematic Reviews and Meta-analyses statement (14) and the Cochrane Handbook for Systematic Reviews of Intervention (15), except for protocol registration.

#### Inclusion criteria

All types of studies conducted on patients undergoing any orthopedic surgical procedure wherein patients were to receive a non-pharmacological intervention for postoperative pain management with or without a control group were included. The study aimed to assess pain outcomes using a validated measurement index, such as the Visual Analog Scale (VAS). Studies using non-pharmacological interventions with regional anaesthesia were excluded. Studies published in a language other than English, case reports, conference abstracts and review articles were also excluded. Conference abstracts were not included in the present study due to incomplete information available from such abstracts. The entire methodology was not clear and according to our experience, in the majority of cases, no response was received when contacting the corresponding authors for clarifications or for missing data.

#### Data extraction and outcomes

The two authors (MF and ZC) independently performed the extraction of available data using the pre-tested standardized format. Details extracted were as follows: First author, publication year, study design, country, demographic details, orthopedic surgery performed, intervention arms, sample size, assessment schedule, tools and interpretations, and results.

#### Quality assessment of studies and meta-analysis

The quality of randomized controlled trials (RCTs) was assessed using the Cochrane Collaboration risk assessment tool (15). The risk of bias in non-randomised studies of interventions (ROBINS-I) tool was used for quality assessment of non-randomized studies (16). Due to considerable heterogeneity in the interventions amongst the included studies, along with the difference in study types, a meta-analysis was not conducted and results are presented in a descriptive fashion.

## Results

### 

#### Selection of studies

**Figure 1:**
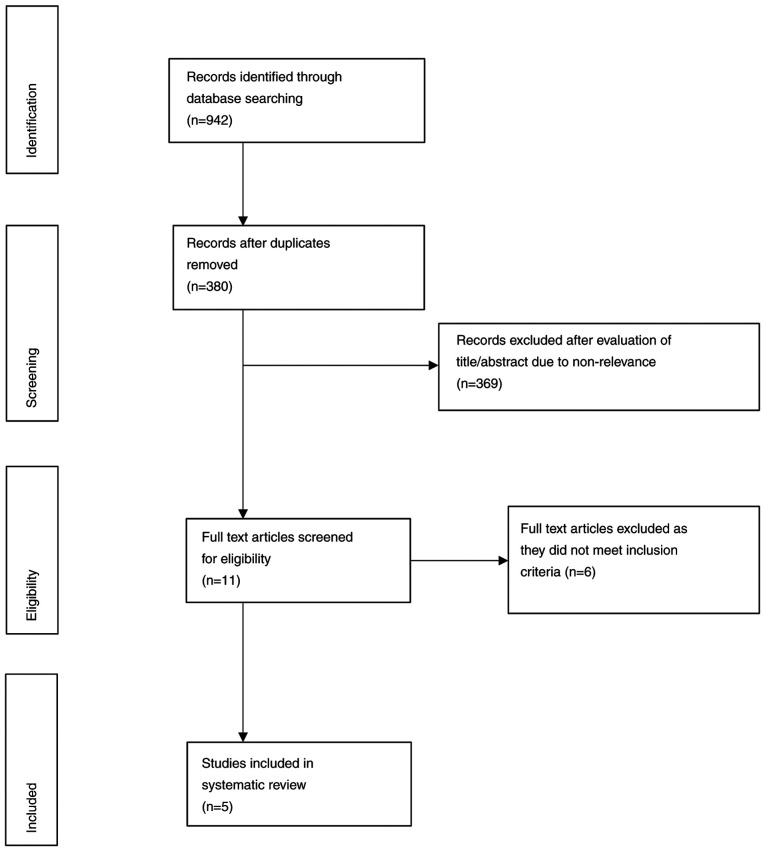
Flow chart for the search and selection of studies.

A flow chart depicting the study retrieval and selection process is presented in Fig. 1. Of the relevant studies identified, five studies were finally included in the present review (17-21) and six studies were excluded (22-27). Details of included studies are presented in Table I and a list of excluded studies with reasons is presented in Table II. A total of three studies were RCTs (17,18,21), while two were non-randomized single-arm studies (19,20). All studies were performed at a single centre. The patient population in the included studies was not coherent. In addition, different non-pharmacological interventions were used in the included studies, including relaxation therapy, guided imagery, music and audio-visual distraction. A detailed description of each study is presented below.

#### Descriptive analysis of RCTs

Büyükyilmaz and Aşti (17), in an RCT on patients undergoing total hip or knee arthroplasty, assessed the role of relaxation techniques and back massage in reducing pain scores. They randomized their sample into intervention and control arms with 30 patients in each group. The relaxation techniques included rhythmic respiration, muscle relaxation exercises and listening to music. Patients also received a 10-min back massage lying on the intact hip/knee joint in a lateral position on their bed. The pain was measured before, immediately after, and one hour and two hours after therapy. The study reported a statistically significant reduction of pain in the intervention arm as compared to the control arm at all follow-up time-points.

Charette *et al* (18), in an RCT on patients who had received spinal fusion, evaluated the role of guided imagery and relaxation exercises in reducing pain scores. A total of 40 adolescent patients were randomly divided into two groups of 20 each. Patients in the intervention arm were provided with a DVD with information and guided imagery/relaxation exercises to practice at least three times a week at home. Pain scores were recorded pre-operatively, as well as at 14 and 30 days post-operatively. The authors reported a statistically significant reduction of pain at all follow-up time-points in the intervention group.

In another RCT, Elmali and Balci Akpinar (21) evaluated the effect of video distraction in reducing post-surgical pain scores in a sample of patients after orthopedic surgery. They randomized their sample into three groups of 30 patients each. The first group watched a comedy video, the second group watched an non-comedy video, while the third group served as a control. Pain scores were recorded on the VAS immediately and 30 min after the intervention. The authors reported a significant reduction of pain scores with the use of both comedy and non-comedy videos, while no such effect was seen in the control group. Pain reduction in both intervention groups indicated that video distraction rather than the comedy element may have served a role in the reduction of pain. Also, lack of blinding may have introduced bias in the overall results.

#### Descriptive analysis of non-RCTs

Schneider (20) conducted a single-arm study on 65 patients who had undergone varying orthopedic procedures. All patients listened to instrumental piano music for 35 min. The music track selection was performed by a researcher who had a strong musical background and formal musical education. Pain scores were recorded prior to and just after the intervention. The authors reported a statistically significant reduction of pain scores after the intervention compared to pre-intervention levels.

Lim *et al* (19), in a single-arm study, assessed the efficacy of relaxation therapy in reducing pain in a cohort of patients with total knee replacement surgery. A total of 18 patients were included in their study. The intervention consisted of three 1-h long individual-based sessions. All patients were first counseled about the negative effects of emotional tension and physical pain on postoperative recovery and the benefits of practicing the relaxation techniques. Following patient education, all patients practiced relaxation therapy consisting of breathing exercises with a background of soothing music and guided imagery. Pain scores were recorded prior to and after the intervention. The authors reported significantly reduced pain with the use of the intervention.

#### Quality of studies

**Figure 2:**
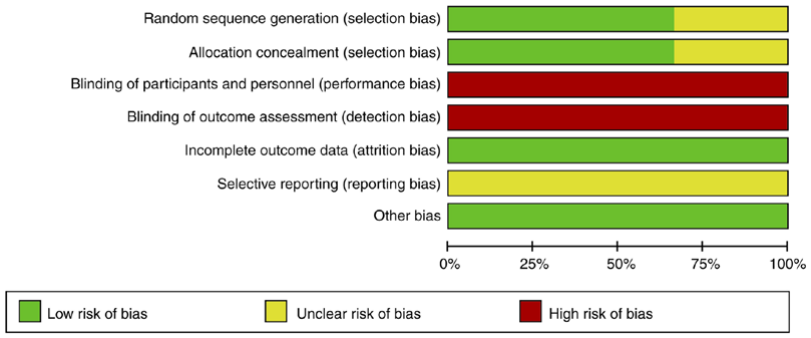
Overall risk of bias summary of randomized controlled trials.

The results of a quality assessment of the included studies are presented in Table III. A total of two RCTs (18,21) had a low risk of bias due to randomization and allocation concealment. Due to the nature of the intervention, blinding was not possible. None of the trials was pre-registered. The summary risk of bias of the RCTs is presented in Fig. 2. The overall quality of non-RCTs was not high with high/unclear risk of bias across multiple domains (Table III).

## Discussion

The present systematic review assessed evidence on the use of non-pharmacological interventions for pain management in patients undergoing orthopedic surgical procedures. The results of the present review indicated that several different strategies of non-pharmacological interventions have been used in orthopedic patients and all such complementary therapies may have certain benefits in the reduction of post-operative pain. The results are to be interpreted with caution, as only a small number of studies with considerable heterogeneity were included.

Non-pharmacological methods of pain management have gained popularity in the past decade owing to their ease of use and the side-effects associated with pharmacological interventions. The most commonly used pain medications after any surgical procedure are NSAIDs and opioids (5). Adverse effects of NSAIDs include gastric ulcers, bleeding complications and kidney injury (5). Specifically in orthopedic practice, the use of NSAIDs may inhibit fracture healing (28). Opioids, on the other hand, may have adverse effects including physical dependence, tolerance, respiratory depression, vomiting and constipation (29). In this context, it is important for clinicians as well as nursing personnel to identify non-pharmacological methods of pain management.

For the present review, five studies assessing different non-pharmacological techniques of pain control after orthopedic surgery were identified and included. A total of three of the studies utilized relaxation therapy for pain control. It has been demonstrated that relaxation therapy is able to provide pain relief by decreasing anxiety, lowering muscle tension and distracting the patient (30). In a recent systematic review and meta-analysis of 12 studies, Ju *et al* (31) revealed that in patients undergoing abdominal surgery, relaxation therapy may achieve better pain relief as compared to standard treatment. In the present review, all three studies reported pain reduction with the use of relaxation therapy. It is important to note that out of the three studies, one was a single-arm study. In the absence of a control group, the actual beneficial effect of relaxation therapy in reducing pain cannot be validated. Also in the trial of Charette *et al* (18), guided imagery was used in combination with relaxation exercises. In guided imagery, mental images of pleasant sights, smells, sounds, tastes or other somatic sensations are used to generate a positive cognitive and emotional state in the patient (32). Due to the limited number of studies and the use of other non-pharmacological techniques with relaxation therapy, strong conclusions on the exact role of relaxation in the management of patients with orthopedic surgery cannot be drawn.

In two studies included in the present review, music and audiovisual distraction were used for pain management. The use of music or audio-visual media is one of the easiest distraction techniques and has been used in various medical disciplines. Song *et al* (10) performed a meta-analysis of nine RCTs, demonstrating significantly reduced pain with the use of music in patients undergoing minor surgical procedures. The use of audio-visual aids has been reported for pain management of pediatric dentistry patients (33), those with sickle cell disease (34) and patients undergoing colonoscopy (11). The trial of Elmali and Balci Akpinar (21) demonstrated that the use of comedy as well as non-comedy videos significantly reduced pain scores in patients following orthopedic surgery. On the other hand, the single-arm study of Schneider (20) also concluded that instrumental piano music may be helpful in reducing pain in the post-operative period.

The present review should be interpreted with the following limitations. First, only five studies were available for inclusion in the present review. Furthermore, there was significant heterogeneity amongst the included studies with respect to the patient population and the non-pharmacological intervention. This limited the feasibility of pooling the data for a meta-analysis. In addition, only three of the included studies were RCTs, while the other two were single-arm studies. Lack of a control group severely limits the ability to draw conclusions regarding the effectiveness of an intervention. As another limitation, the overall quality of studies assessed by the Cochrane tool and ROBINS-I tool was not high. There were numerous sources of bias in the included studies, for example lack of blinding, which weakens the conclusions that may be drawn from these trials. Finally, the studies included in the present review were restricted to those published in the English language, owing to constraints of translation. Furthermore, the present review did not include any studies published only as conference abstracts, owing to the limited data available from such publications.

To conclude, the present systematic review analyzed the role of non-pharmacological interventions in pain management provided to patients after orthopedic surgery. Current evidence from a limited number of studies indicates that there may be a potential role of non-pharmacological interventions, including relaxation therapy, guided imagery, music and audio-visual distraction, in the pain management of patients with orthopedic surgery. Owing to the considerable heterogeneity and risk of bias in the included studies, strong conclusions cannot be drawn. Further high-quality RCTs assessing the role of such non-pharmacological techniques of pain management are required to strengthen the current evidence.

## Supplementary Material

Table SI. Search strategy.

## Figures and Tables

**Table I:** Characteristics of included studies.

First author (year)	Study design	Country	Sex/age (years)	Orthopedic surgery	Intervention arms	Sample size	Assessment schedule/Assessment tool/ interpretation	Results	(Refs.)
Elmali (2017)	RCT	Turkey	69% male, 31% female/mean,	Arthroscopy, implant removal, osteotomy,	20 min comedy video + standard care	30	Before, immediately after, 30 min after	Statistical significant reduction of pain scores	(19)
			37.41+14.82	hallux valgus surgery, mass excision	20 min non-comedy video + standard care	30	intervention/Visual Analogue Scale/0=no	in both intervention groups with no pain	
					Standard care	30	pain, 100=as bad as it could be	reduction in the control group.	
Schneider (2018)	Single-arm study	US	79% male, 21% female/mean, 61.75	Total hip replacement, fractured hip repair, repair of upper extremity fracture, ankle fractures	35 min individual music therapy with CD player and headphones	65	Before, after intervention/Visual Analogue Scale/1=least intense pain, 10=most intense pain	Statistically significant reduction of pain after listening. to music	(20)
Lim (2014)	Single-arm study	Singapore	28% male, 72% female/range, 21-75	Total knee hip replacement	1 h/session breathing exercises, muscle relaxation, guided imagery; total 3 sessions	18	Before, after intervention/Numerical Pain Rating Scale/0=no pain, 10=unbearable pain	Statistically significant reduction of pain with the intervention	(21)
Charette (2015)	RCT	Canada	18% male, 72% female/mean, 15+2.15	Post spinal fusion	CD with information and guided imagery plus relaxation exercises, 3x/week at home + standard care	20	Before surgery, day of discharge, 14 days post-op, 30 days post-OP/Brief Pain Inventory/0=no pain; 10=worst possible pain	Significant reduction of pain scores in the intervention group at all follow-up time points.	(18)
					Standard care	20			
Büyükyilmaz (2013)	RCT	Turkey	30% male, 70% female/mean (range), 58.2 (24-83)	Total hip or knee arthroplasty	Relaxation techniques (rhythmic respiration, music relaxation techniques, listening to music, back massage)	30	Before, immediately after, 1 h after, 2 h after intervention/Visual Analogue Scale/1=least	Significant reduction of pain scores in the intervention group at all follow-up time-points.	(17)
					Standard care	30	intense pain, 10=most intense pain		

All studies were single-center studies. RCT, randomized controlled trial; CD, compact disc.

**Table II:** Excluded studies with reasons.

Stud*y*	Reason for exclusion
Chiodo *et al* (27)	Did not evaluate pain outcome
Tolunay *et al* (26)	Study not on orthopedic surgical patients
Athanassoglou *et al* (25)	Use of distraction with regional anesthesia
Hsu *et al* (24)	Did not evaluate pain outcome
Eckhouse *et al* (23)	Did not evaluate pain outcome
Rupérez Ruiz *et al* (22)	Use of distraction with regional anesthesia

**Table III:** Risk of bias in included studies.

A, RCTs							
First author, year (Ref.)	Random sequence generation	Allocation concealment	Blinding of participants and personnel	Blinding of outcome assessment	Incomplete outcome data	Selective reporting	Other bias
Lim 2014(19)	Low risk	Low risk	High risk	High risk	Low risk	Unclear risk	Low risk
Charette 2015(18)	Low risk	Low risk	High risk	High risk	Low risk	Unclear risk	Low risk
Büyükyilmaz 2013(17)	Unclear risk	Unclear risk	High risk	High risk	Low risk	Unclear risk	Low risk
B, Non-RCTs							
Study	Bias in selection of participants	Bias due to confounding	Bias in classification of intervention	Bias due to deviations from intended interventions	Bias due to missing data	Bias in measurement of outcomes	Bias due to selective outcome reporting
Schneider 2018(20)	High risk	Unclear risk	Low risk	Unclear risk	Low risk	High risk	Unclear risk
Lim 2017(21)	Unclear risk	Unclear risk	Low risk	Unclear risk	Low risk	High risk	Unclear risk

RCT, randomized controlled trial.

## Data Availability

The datasets used were from published studies.
