# Supplementary material for: A systematic review of non-pharmacological interventions used for pain relief after orthopedic surgical procedures
Source: Exp Ther Med. 2020 Sep 1;20(5):36. doi: 10.3892/etm.2020.9163 (PMC7480131; doi:10.3892/etm.2020.9163)
Supplement: Table SI. Search strategy. [file Supplementary_Data.pdf]

Table SI. Search strategy.

Search ID	Features/search terms
1	exp Orthopedics/
2	‘wounds and injuries’/ or arm injuries/ or athletic injuries/ or crush injuries/ or joint dislocations/or fractures, bone/ or hand injuries/ or hip injuries/ or leg injuries/ or neck injuries/ or shoulder injuries/ or spinal cord injuries/ or ‘sprains and strains’/ or thoracic injuries/
3	fracture.mp. or exp Fracture Fixation, Intramedullary/ or exp Fracture Fixation, Internal/ or exp Open Fracture Reduction/ or exp Fracture Fixation/ or exp Fracture Healing/
4	(bone adj1 fracture).mp.
5	or/1-4
6	exp Fentanyl/ or exp Analgesics, Opioid/ or opiod.mp. or exp Analgesics/
7	exp Narcotics/
8	exp Anti-Inflammatory Agents, Non-Steroidal/
9	video.mp. or exp Video-Audio Media/
10	exp Music Therapy/ or exp Music/ or music.mp.
11	guided imagery.mp. or exp ‘Imagery (Psychotherapy)’/
12	muscle relaxation.mp. or exp Muscle Relaxation/
13	breathing exercise.mp. or exp Breathing Exercises/
14	or/6-13
15	5 and 14
16	Randomized Controlled Trial/
17	Controlled Clinical Trial/
18	(Nonrandom* or non-random* or non-random* or quasi-random* or quasirandom*).ti,ab,hw,kf,kw.
19	Control Group/
20	[(quasiexperimental or quasi-experimental) adj3 (study or studies or trial*)].ti,ab,hw,kf,kw.
21	systematic review.mp. or exp ‘Systematic Review’/
22	or/16-21
23	15 and 22
24	limit 23 to (English language and humans)
25	(article in press or case report or chapter or comment or conference abstract or conference paper or conference review editorial or letter or note or review or short survey).pt.
26	24 not 25
